# *PLOS Genetics* 2018 Reviewer and Editorial Board Thank You

**DOI:** 10.1371/journal.pgen.1008031

**Published:** 2019-02-27

**Fig 1:**
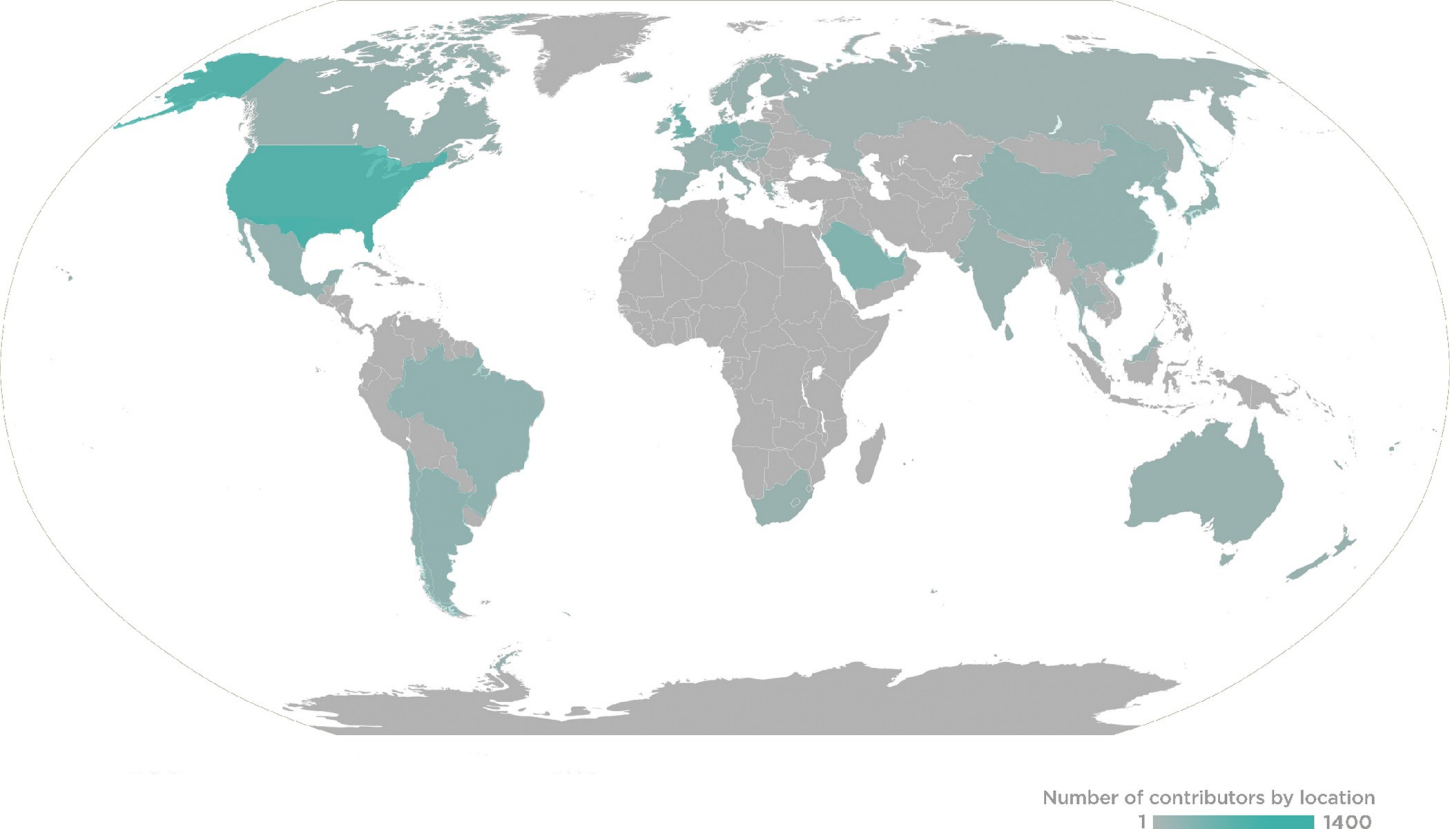
2018 *PLOS Genetics* Global Editor and Reviewer Locations.

PLOS and the *PLOS Genetics* team want to sincerely thank all of our Editorial Board Members, Guest Editors, and Reviewers for the journal in 2018. Your contributions of time and expertise support your research community, advance scientific progress, and continue to make *PLOS Genetics* a leader in its field. This past year, *PLOS Genetics* received the assistance of 183 Editorial Board members, 349 Guest Editors, and 2,339 Reviewers, who handled 2,175 manuscripts that resulted in 581 publications (Fig 1).

We’re deeply grateful to all of our volunteers whose dedicated efforts support *PLOS Genetics* and Open Science. Thank you all for your work!
